# Adolescent abortions: Situational analysis based on official statistics conducted in Kazakhstan during the last 5 years (2007–2011)

**DOI:** 10.5195/cajgh.2013.38

**Published:** 2013-10-01

**Authors:** Gulya Nazarovna Alimbayeva, Gulzhan Narimbayevna Chimbayeva

**Affiliations:** 1 Kazakh National Medical University, Almaty, Kazakhstan; 2 Hospital of the President Administration, Astana, Kazakhstan

**Keywords:** abortion, adolescent pregnancies, lethal outcomes, reproductive age, statistics database

## Abstract

In recent decades, adolescent pregnancy has become an important health issue in a great number of both developed and developing countries. We have investigated the official statistics database of the National Ministry of the Health (MoH) and their #13 Statutory Form (SF) and found the total number of abortions between 2007–2011 in Kazakhstan decreased by 28%. The total number of adolescent (up to 15 plus 15–18 years old) abortions decreased by 52.7%. Contrary to this decrease in the total number of adolescent abortions, spontaneous abortions have increased from 23.2% to 45.0%,. We found a tendency towards a decrease in the number of adolescents with the first pregnancy among adolescents 15–18 who had abortion between 2007–2011, from 51.3% to 35.8%. This clearly reflects the success of prevention activities among adolescents who have already had an abortion or child labor. During the analyzed period, there were two lethal outcomes from abortionsamong girls15–18 years old. There are some limitations in the assessment levels and dynamic changes of abortions among adolescents due to the division of age in the official statistical database.

## Introduction

The World Health Organization’s (WHO) definition of health, “Health is a state of complete physical, mental and social well-being and not merely the absence of disease or infirmity” was adopted on 7 April 1948 and has not been amended.^1^ But what is an unwanted pregnancy and abortion? Is it health or illness?

In recent decades, adolescent (<20 years of age) pregnancyhas become an important health issue in a number of developed and developing countries. However, pregnancy during adolescence is by no means a new phenomenon. In many countries, large numbers of adolescent pregnancies and births are reported. Two decisive aspects of the adolescent period have strongly influenced this increase. The first is the decreasing age at menarche and the second is the increasing age of marriage. This widening gap is the basis of unwanted pregnancies during young ages.

Why is the issue of adolescent pregnancy so difficult to solve? If we investigate the definitions that cover this specific life period, we can determine that:

- According to the UN Convention on the Rights of the Children,^2^ a child is an individual below the age of 18.
- Adolesence is defined by WHO^3^ as individuals of 10–19. The WHO & Adolescent Health Care divides adolescence into three psychosocial developmental phases:
  1. Early adolescence: age of 10–13 years old
  2. Middle adolescence: age of 14–16 years old
  3. Late adolescence: age of 17–19 years old
- Reproductive age for females: 15–44 years old.^4^

Pregnancy is a great burden for adolescents. A female in this age range may be simultaneously treated as a child, an adolescent, and a reproductive female. We can determine the causes of the increase in adolescent pregnancies if we consider their social backgrounds and that pregnancies may happen in children with reproductive potential.

## Methods

To gain an understanding of the issue of adolescent pregnancies in Kazakhstan during the period of 2007–2011, we have investigated the official statistics database of the National Ministry of the Health (MoH) and their # 13 Statutory Form (SF). We first investigatedabortion. The # 13 (SF) has long been in use in Kazakhstan.^5^ There are some limitations in the assessment levels and dynamical changes of the abortions among adolescents because databases are divided into several columns (e.g. less than 15 years of age, ages 15–18 and 19 and above).

## Results

There was a 28% reduction in the number of abortions between 2007 and 2011 in Kazakhstan. On the other hand, the total number of adolescent (up to 15 plus 15–18 years old) abortions decreased by 52.7%. This happened as the result of prevention activities of medical specialists and social programs.

**Figure 1:**
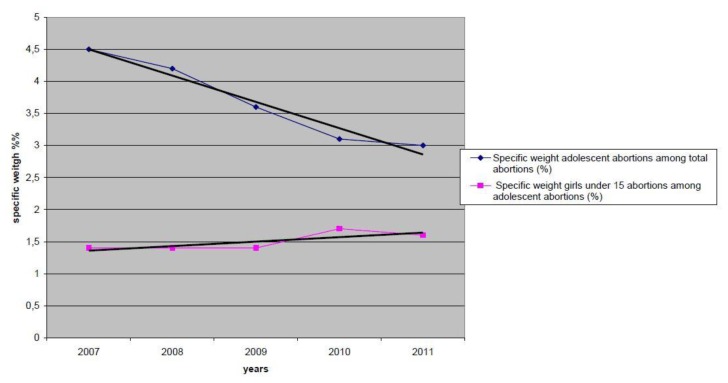
Dynamic changes of the specific weight of adolescent abortions compared to total amount of abortions during the period 2007–2011

Figure 1 shows the dynamics of reduction for the specific weight of adolescent abortions among the total number of abortions. This figure illustrates a decreasing trend from 4.5% to 3.0%. Contrary to the specific weight of adolescent abortions, the figure also indicates that abortions in adolescents under age 15 remains constant, with slight increase from 1.4%–1.6%.

**Figure 2:**
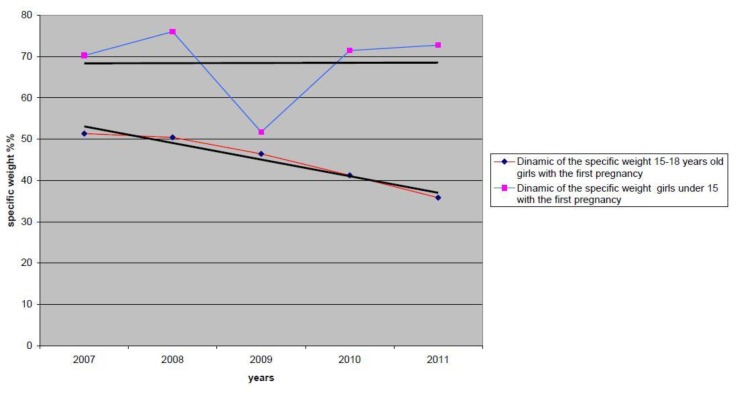
Dynamic of the specific weight adolescents with the first pregnancy among adolescents who had abortion during 2007–2011

Figure 2 illustrates a decreasing trend in the specific number of first pregnancy abortions among adolescents ages 15–18, from 51.3% to 35.8%. This fact clearly reflects the effectiveness of prevention activities among adolescents who have already had abortion or labor.

**Figure 3:**
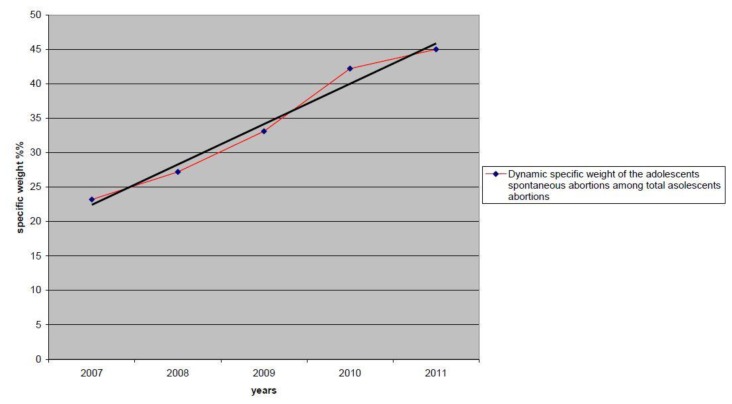
Dynamic of specific weight of adolescents spontaneous abortions among total adolescents abortions during the period 2007–2011

Against the background of a decrease in the total number of adolescent abortions, specific weight of spontaneous abortions has increased from 23.2% to 45.0%. This is difficult to explain and warrants further investigation. Figure 3 illustrates this trend.

During the analyzed period, there were two lethal outcomes from spontaneous abortions among females ages 15–18 (1 in 2008 and 1 in 2010). Abortion lethality in adolescents reached 0.02% in 2008 and 0.03% in 2010. At the same time, the rate of total abortion lethality in Kazakhstan has never exceeded 0.01%. This statistically confirmed database underlines the particular danger of unwanted pregnancy among adolescents. Data on regional specifics of adolescent abortions were not available.

## Conclusion

This paper provides an overview of statistics on adolescent abortions, which is a serious public health concern in Kazakhstan. Despite modest improvements, adolescent abortions in Kazakhstan remain burdensome for medical specialists and teachers, calling for the need to establish more effective public health programs to reduce the number of abortions in young and vulnerable population groups. Adolescents who have had a previous abortion are at a higher risk of subsequent procedures as compared to those who have never had an abortion. Considering that there were two deaths associated with abortions in 2008 and 2010, adolescents must be better educated about reproductive health. Our paper demonstrates wide disparities in younger vs. older adolescent groups, with lesser success achieved in adolescents under the age of 15. Methods that are currently used for reduction of the total number of adolescent abortions in Kazakhstan need improvement, especially among females under 15 years of age.
